# Barriers to screening, diagnosis and management of hyperglycaemia in pregnancy in Africa: a systematic review

**DOI:** 10.1093/inthealth/ihab054

**Published:** 2021-08-25

**Authors:** Thomas Hinneh, Albrecht Jahn, Faith Agbozo

**Affiliations:** Dormaa East District Hospital, Department of Nursing, P.O. Box 38, Wamfie, Ghana; Heidelberg Institute of Global Health, Heidelberg University, Heidelberg 69120, Germany; Heidelberg Institute of Global Health, Heidelberg University, Heidelberg 69120, Germany; Heidelberg Institute of Global Health, Heidelberg University, Heidelberg 69120, Germany; University of Health and Allied Sciences, Department of Family & Community Health, Private Mail Bag 31, Ho, Ghana

**Keywords:** Africa, diagnostic tests, gestational diabetes, maternal health services, pregnant women, treatment

## Abstract

Gestational diabetes mellitus (GDM) complicates pregnancies in Africa. Addressing the burden is contingent on early detection and management practices. This review aimed at identifying the barriers to diagnosing and managing GDM in Africa. We searched PUBMED, Web of Science, WHOLIS, Google Scholar, CINAHL and PsycINFO databases in May 2020 for studies that reported barriers to diagnosis and management of hyperglycaemia in pregnancy. We used a mixed method quality appraisal tool to assess the quality and risk of bias of the included studies. We adopted an integrated and narrative synthesis approach in the analysis and reporting. Of 548 articles identified, 14 met the eligibility criteria. Health system-related barriers to GDM management were the shortage of healthcare providers, relevant logistics, inadequate knowledge and skills, as well as limited opportunities for in-service training. Patient-related barriers were insufficient knowledge about GDM, limited support from families and health providers and acceptability of the diagnostic tests. Societal level barriers were concomitant use of consulting traditional healers, customs and taboos on food and body image perception. It was concluded that constraints to GDM detection and management are multidimensional. Targeted interventions must address these barriers from broader, systemic and social perspectives.

## Introduction

Across the globe, diabetes contributes significantly to the burden of non-communicable diseases (NCDs).^1^ According to the 2015 International Diabetes Federation report, among the 15.2% of pregnancies affected by hyperglycaemia in pregnancy (HIP) globally, gestational diabetes mellitus (GDM) constituted 85% of all cases.^2^ Even though the current GDM epidemic affects both high- and low-income countries, it is estimated that nearly 90% of the global cases occur in low-income countries.^3^ Pathologically, over 50% of pregnant women who develop GDM progress to develop type II diabetes within 2–10 y after the index diagnosis.^4,5^ If left unchecked, GDM might compound the already high burden of NCDs in the African region, where about 80% of the cases of diabetes occur.^1,6,7^

In accordance with the WHO guideline, GDM is defined as carbohydrate intolerance resulting in hyperglycaemia of variable severity, with onset or first detection in pregnancy.^8^ Even though diabetes in pregnancy (known diabetes before pregnancy) is a matter of concern, the most common type of HIP is GDM^3^ and, therefore, understanding the practices associated with screening, diagnosis and management within the African context is essential.

There is ample evidence that GDM exposes pregnant women to the risk of caesarean section, traumatic delivery, prolonged delivery, pregnancy-induced hypertension, pre-eclampsia and could also lead to maternal and foetal death.^9,10^ Evidence has also shown that exposure of the foetus to a hyperglycaemic intrauterine environment increases the risk of macrosomia, anencephaly, spinal bifida, cerebral palsy and large for gestational age.^9,11–14^ Apart from these short-term complications, offspring born to mothers with GDM are also at a higher risk of developing diabetes and obesity in later life.^1,15,16^

Glycaemic control through lifestyle modification and medical therapy during pregnancy are promising strategies to reduce the risk of adverse foetal, perinatal and neonatal events.^17–19^ However, preventing complications and improving disease prognosis hinge on early detection and effective management. Nonetheless, some studies have established the merits of universal screening for GDM.^20,21^ However, practical implementation of detection and management approaches may not be feasible due to multiple constraints. Therefore, identifying these factors would be essential in informing policy decisions on addressing the burden of GDM.

In this systematic review, we performed a comprehensive literature search to summarise evidence regarding the barriers to screening, diagnosing and managing GDM in Africa. We sought to answer three research questions: (1) What impedes GDM screening and testing? (2) What are the barriers towards supporting and managing pregnant women diagnosed with GDM? (3) How can the experiences of pregnant women regarding GDM testing and management help to improve care?

## Methods

### Study design

We conducted this systematic review by searching through PUBMED, Web of Science, WHOLIS, Google Scholar, CINAHL and PsycINFO databases, taking into consideration the guideline of ‘Preferred Reporting Items for Systematic Reviews and Meta-analyses’ (PRISMA) statement.^22^ A protocol for the review was developed a priori and registered with the PROSPERO international register for systematic reviews (2020: CRD42020180335).^23^ Using the PICo framework recommended by the Joana Briggs Institute (JBI) and Cochrane collaboration as the preferred approach for developing review questions, search terms for the review were categorised into three components: P=Population, I=Phenomenon of Interest and Co=Context. Where appropriate, Medical Subject Heading (MeSH) terms were used. The search terms used and the search strategy for each database are included (Supplementary Data 1). The review started in February 2019, but the search was completed in May 2020. Eligible studies included in this review were published from 2012 to 2019 in English, which captured recent challenges to GDM care in the Africa region. Reference lists of included studies were also screened for eligible studies.

### Inclusion and exclusion criteria

Studies considered for inclusion were peer-reviewed, published, quantitative, qualitative, mixed-methods and randomised research papers conducted in any African country or subregion related to barriers to screening, diagnosis or management of GDM and/or diabetes in pregnancy (DIP) that focused on the women and their families, as well as health practitioners, policymakers and stakeholders involved in the care process. Studies that focused exclusively on prevalence and risk estimation or involved postpartum women were excluded.

### Study selection and eligibility

We included qualitative, quantitative and mixed-method studies. Deduplication, title and abstract screening, and reviewing of reference lists of potentially eligible studies for relevant literature and full-text screening, was performed by TH. FA also independently screened the full-text articles to assess their eligibility. In instances where a decision could not be reached, discussions were held with AJ. All studies retrieved were imported to Endnote library where deduplication was performed using Barmer's method.^23^

### Data handling and extraction

Data was extracted by TH using Microsoft Office Excel. In addition to the findings of the studies, other data extracted included the names of authors, aim of the study, year of publication, geographic zone where the study was conducted, study design, sampling methods, sample size, characteristics of participants, gestational age of participants at the time of screening and level of healthcare where the study was conducted. TH and FA independently reviewed the data extracted for each study using the data items listed in the review protocol to ensure that the data extracted were compliant with the review objectives.

### Risk of bias and quality assessment

Quality assessment of studies included in this review were independently assessed by TH using a mixed-method quality appraisal tool (MMAT).^24^ FA reassessed a subset of the studies (one in each design category) to verify the appraisal outcomes. The studies were initially subjected to two mandatory screening questions according to the MMAT tool. A ‘yes’ answer was obtained for all of the studies, making it feasible to apply the subsequent questions based on the study's design. Overall, the scores obtained as per methodological criteria and quality assessment ranged from 2 to 5 out of a total possible score of 5. A mark of 5 (represented by five asterisks [*****]) implied that the study met 100% of the quality criteria, whereas marks of four (****), three (***), two (**) and one (*) corresponded to 80%, 60%, 40% and 20% of the quality criteria, respectively (Table 1). Overall, the studies were of appreciable quality with the final quality rating ranging from 60 to 100%. Qualitative studies incorporated in the review showed adequate interpretation of results supported by specific quotations from respective participants. There was adequate coherence between data collected and the interpretation of findings. Three of the qualitative studies^25–27^ used data saturation as a sample size determination approach. One common trend observed in the mixed-method studies was the inadequate integration of qualitative and quantitative data sources.

**Table 1.** Result of quality and risk assessment using the mixed method appraisal tool^24^

Studies	Q 1.1	Q 1.2	Q 1.3	Q 1.4	Q 1.5	Q 2.1	Q 2.2	Q 2.3	Q 2.4	Q 2.5	Q 4.1	Q 4.2	Q 4.3	Q 4.4	Q 4.5	Q 5.1	Q 5.2	Q 5.3	Q 5.4	Q 5.5
Mensah et al., 2017^31^	1	1	1	1	1															
Mensah et al., 2019^27^	1	1	1	1	1															
Mukona et al., 2017a^32^	1	1	1	1	1															
Mukona et al., 2017b^25^	1	1	1	1	1															
Muhwava et al., 2019^33^	1	1	1	1	1															
Muhwava et al., 2018^26^	1	1	1	1	1															
Woticha et al., 2019^34^	1	1	1	1	1															
Ugboma et al., 2012^35^						1	1	1	0	1										
Nwose et al., 2019^29^											1	0	1	0	1					
Njete et al., 2018^30^											1	1	1	1	1					
Utz et al., 2016^39^	1	1	1	1	1						1	0	1	1	1	1	1	1	1	1
Nielsen et al., 2012^37^*	1	1	1	1	1						1	0	1	1	1	1	1	1	1	1
Nielsen et al., 2012^38^*	1	0	1	0	1						1	1	1	1	1	1	1	1	1	1
Mukona et al., 2017^36^*	1	1	1	0	0						1	1	1	1	1	0	1	1	1	1
**Questions for the quality rating**																				
**(Qualitative studies)**									**(Quantitative descriptive studies)**											
Q 1.1. Is the qualitative approach appropriate to answer the research question?									Q4.1. Is the sampling strategy relevant to address the research question?											
Q 1.2. Are the qualitative data collection methods adequate to address the research question?									Q 4.2. Is the sample representative of the target population?											
Q 1.3. Are the findings adequately derived from the data?									Q 4.3. Are the measurements appropriate?											
Q 1.4. Is the interpretation of results sufficiently substantiated by data?									Q 4.4. Is the risk of non-response bias low?											
Q 1.5. Is there coherence between qualitative data sources, collection, analysis and interpretation?									Q 4.5. Is the statistical analysis appropriate to answer the research question?											
									Q 5.1. Is there an adequate rationale for using a mixed methods design to address the research question?											
**(Randomised control trial studies)**									**(Mixed method studies)**											
Q 2.1. Is randomisation appropriately performed?									Q 5.2. Are the different components of the study effectively integrated to answer the research question?											
Q 2.2. Are the groups comparable at baseline?									Q 5.3. Are the outputs of the integration of qualitative and quantitative components adequately interpreted?											
Q 2.3. Are there complete outcome data?									Q 5.4. Are divergences and inconsistencies between quantitative and qualitative results adequately addressed?											
Q 2.4. Are outcome assessors blinded to the intervention provided?									Q 5.5. Do the different components of the study adhere to the quality criteria of each tradition of the methods involved?											
Q 2.5 Did the participants adhere to the assigned intervention?																				

Key: yes (1 point), no (0 points), cannot tell (0 points).

*Mixed method studies; NB: questions ranging from 3.1 to 3.4 are missing as none of the studies adopted a quantitative non-randomised approach. Studies are arranged in order of the design; the first five studies are qualitative, the next one is a randomised controlled trial, the next two are quantitative and the last four are of mixed method design.

A quality rating of ***** means that 100% quality criteria were met, **** 80%, *** 60%, ** 40% and * 20%.

### Data synthesis

**Figure 1.**
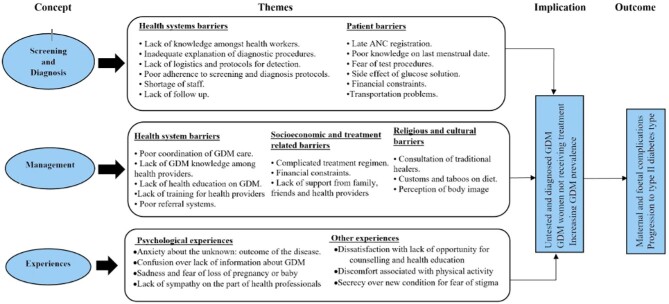
Thematic synthesis of results showing a summary of the themes and subthemes. ANC, antenatal care.

Given the heterogeneity of the design of articles included in the review, we followed an integrated and narrative synthesis approach described by the JBI mixed method systematic review methodology.^28^ The integrated synthesis approach allowed combining data extracted from quantitative and qualitative studies for further analysis into themes. The quantitative studies^29,30^ were ‘qualitised’ using textual descriptions of the findings. TH and FA read and re-read the full text of the studies to understand how the barriers to GDM care were reported in the articles. Following the grouping of the articles according to the three specific objectives, we performed a thematic analysis of the results. We then discussed the themes generated until agreements on the themes were reached. The major themes generated were health system- and patient-related barriers under objectives one and two. An additional subtheme on sociocultural barriers was generated specifically under objective two. The themes and subthemes are summarised and shown in Figure 1.

## Results

### Search outcome

**Figure 2.**
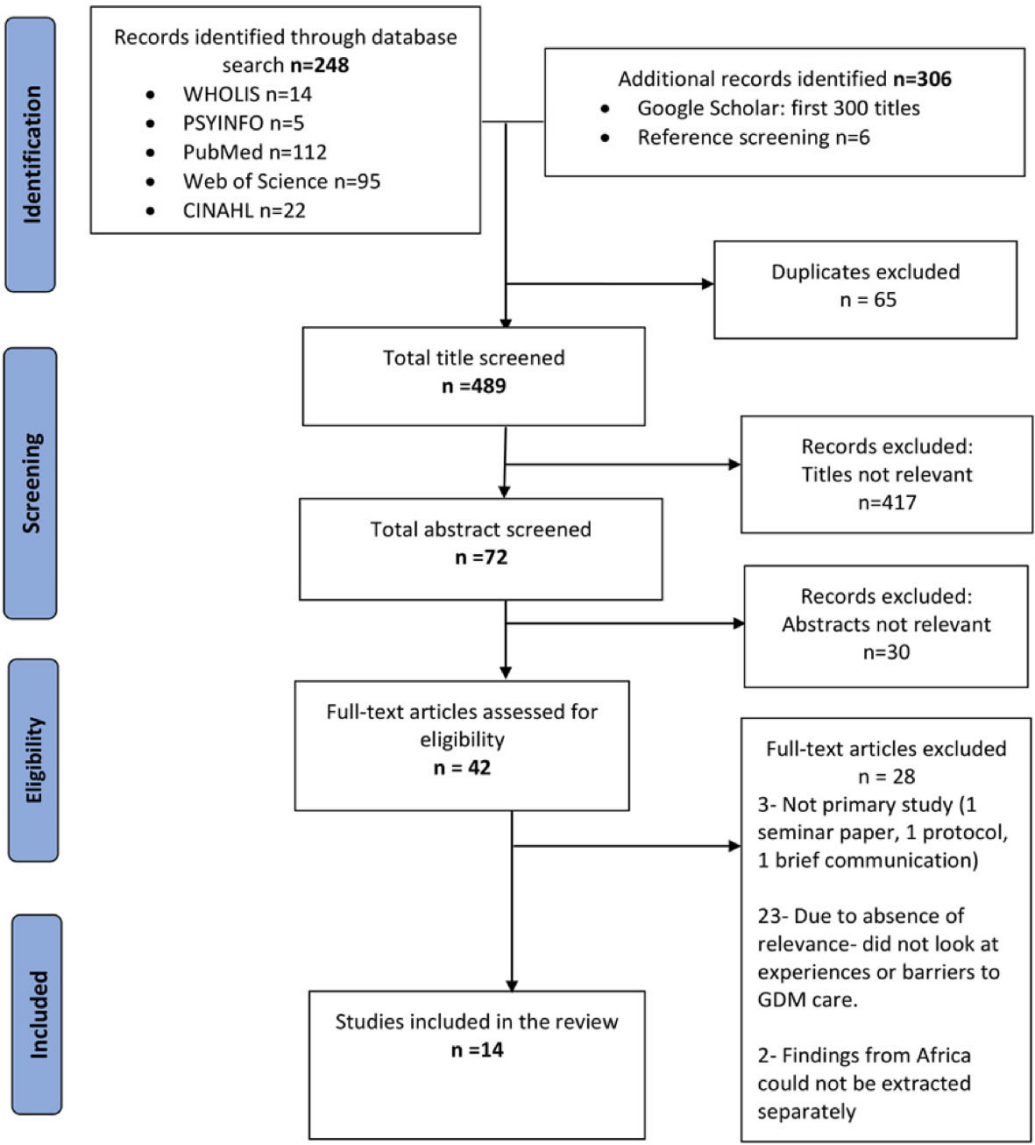
Prisma flow diagram illustrating studies included during the selection process and the reasons for the exclusions.

The search of the various electronic databases and other sources yielded 548 articles. Sixty-five duplicates were found and removed; 489 articles had their titles screened, of which 417 were excluded. The remaining 72 had their abstracts screened, and 30 other studies conducted outside Africa were removed. Forty-two full-text articles were assessed for eligibility, of which 28 were excluded. Articles were eliminated on the basis of being non-primary research (n=3) alongside not being relevant regarding barriers to GDM care or reporting any experience on GDM screening, diagnosis or management (n=23). Two articles that presented findings from low- and middle-income countries (LMICs) were excluded because findings specific to Africa could not be extracted separately. The study selection process is represented in a PRISMA flow diagram in Figure 2.

#### Studies included in the review

A total of 14 primary studies were included in this review comprising 7 solely qualitative articles, 2 solely quantitative articles, 4 mixed-methods articles and 1 randomised control trial. The overview of the studies included in this review is presented in Table 2. Half of the studies employed qualitative designs,^25–27,31–34^ with interviews and focus group discussions as the predominant approaches for data collection. Two of the studies were quantitative,^29,30^ one was a randomised controlled trial^35^ and four employed mixed-method designs.^36–39^ The sample size of the papers included ranged from 10 to 3080, while the total sample size from the 14 studies was 4006.

**Table 2.** Overview of studies included in the review

Authors/year/country	Focus of the paper	Design and sampling approach	Population and sample size	Age, y (range)	Sampling context	Quality rating
(Mensah et al., 2019)^27^ Ghana	Experience with GDM diagnosis and management	Descriptive phenomenological, purposive sampling, semistructured interviews	10 women with GDM	30–42	Primary hospital	*****
(Muhwava et al., 2019)^33^ South Africa	Experiences of lifestyle changes among GDM women	Qualitative study, in-depth interviews, focus group discussion	30 women with GDM	25–35+	Tertiary hospital	*****
(Nwose et al., 2019)^29^ Nigeria	Barriers to GDM diagnosis	Mixed method study, clinical observational of records and procedures, focus group discussion	^c^119 pregnant women and health professionals	≤34 and ≥35	Tertiary hospital and primary hospital	***
(Woticha et al., 2019)^34^ Ethiopia	Barriers to detection and management of GDM	Qualitative descriptive study, in-depth interviews	18 obstetricians, midwives, nurses and health officers	26–48	Secondary hospital	*****
(Muhwava et al., 2018)^26^ South Africa	Perspectives on the barriers and opportunities for delivering an integrated mother–baby health service	Descriptive study, in-depth interviews	11 key informants	NR	Secondary and tertiary hospitals	*****
(Njete et al., 2018)^30^ Tanzania	Challenges of GDM screening	Multisetting cross-sectional study, purposive sampling	433 pregnant women	15–49	Tertiary hospital Primary health centre	*****
(Mensah et al., 2017)^31^ Ghana	Experience and barriers with nursing management of GDM	Descriptive phenomenological, purposive sampling, semistructured interviews	8 women with GDM, 7 nurse-midwives	Women 28–48, Nurse-midwives 32–50	Tertiary hospital Primary hospital	*****
(Mukona et al., 2017)^32^ Zimbabwe	Barriers and solutions of adherence in antidiabetic therapy in pregnancy: patients’ perspective	Descriptive qualitative study, purposive sampling, focus group discussion	35 women with GDM	19–49	Tertiary hospital	*****
(Mukona et al., 2017)^25^ Zimbabwe	Barriers and facilitators of adherence in antidiabetic therapy in pregnancy: healthcare workers’ perspective	Descriptive qualitative study, purposive sampling with focus group discussion	28 obstetricians, dieticians, midwives and medical doctors	20–60	Tertiary hospital	*****
*(Mukona et al., 2017)^36^ Zimbabwe	Barriers of adherence of antidiabetic therapy in pregnancy	Mixed sequential design done in two phases	I57 women with DIP and 8 health workers	Women 18–44	Not specified	****
*(Utz et al., 2016)^39^ Morocco	Challenges of screening and management of GDM	Descriptive mixed methods, document reviews, exit interviews, focus group discussion	20 informants, 32 pregnant women and 299 files of women diagnosed with GDM	NR	Primary health centre, secondary, tertiary	****
*(Nielsen et al., 2012)^37^ Kenya, Cameroun, Sudan and other LMICs^a^	Barriers to screening and diagnosis of GDM	Mixed methods, questionnaires, semistructured interviews	8 GDM ^c^project partners	NR	GDM projects in selected health facilities	*****
*(Nielsen et al., 2012)^38^ Sudan, Kenya, Cameroon and other LMICs^b^	Barriers to screening, diagnosis and management of GDM	Mixed methods approach using questionnaires and interviews	10 GDM ^c^project partners	NR	GDM project in selected health facilities	****
(Ugboma et al., 2012)^35^ Nigeria	Importance of screening and incidence of undiagnosed GDM	Randomised controlled trial	3080 pregnant women	NR	Tertiary, secondary and primary hospitals	****

Abbreviations: DIP, diabetes in pregnancy; GDM, gestational diabetes mellitus; LMICs, low- and middle-income countries; NR, not reported.

^a^other LMICs, India, Cuba, China.

^b^India, Cuba, Jamaica, China.

^c^project partners for the two projects were healthcare providers, pregnant women and women with a history of GDM.

Studies are arranged in chronological order.

A quality rating of ***** means that 100% quality criteria were met, **** 80%, *** 60%, ** 40% and * 20%.

In terms of geographical location, five studies were conducted in Southern Africa,^25,26,32,33,36^ four in either West^27,29,31,35^ or East Africa,^30,34,37,38^ two in Central Africa^37,38^ and one in North Africa.^39^ In addition, two of the studies were multi-country (Cameroun, Kenya, Sudan),^37,38^ yielding data reported from 16 countries in 14 studies overall.

### Demographic characteristics of participants diagnosed with GDM and DIP

Participants in the eligible studies were women diagnosed with either GDM^26,27,31,33^ or DIP.^32,36^ Other studies generally focused on pregnant women attending antenatal clinics where an adjunct objective was to explore barriers related to GDM screening, diagnosing or management.^29,30,35,39^ The age of the women ranged from 15 to 49 y. Despite the limited evidence on how marital status could influence screening and diagnosis of GDM, one study reported that women who were not in any relationship had a higher chance of not returning for a confirmatory test such as an oral glucose tolerance test (OGTT) and fasting plasma glucose.^30^

### Healthcare context

In Table 2, we provide details of the contexts under which the studies were conducted, thus indicating the level of healthcare at which GDM services are often provided. A key factor that facilitated service provision for the detection and management of GDM was the availability of skilled healthcare providers at various healthcare levels. Utz et al. and Mukona et al. demonstrated that women in Morocco and Zimbabwe were referred to tertiary or advanced levels of healthcare where specialists such as endocrinologists and gynaecologists are stationed. However, the low socioeconomic status of some pregnant women, poor road networks and work schedules could discourage women from accessing healthcare at tertiary levels.^25,32,36,39^ Meanwhile, Utz et al. suggested that decentralising screening, diagnosis and non-pharmacological management of GDM to the primary level of care would improve access and mitigate the risk of complications.^39^ In six of the eight studies included, healthcare providers constituted the study participants. In these studies, obstetricians, nurses, midwives and nurse-midwives were the professionals most frequently involved in the screening and diagnosing of GDM, even at tertiary levels of healthcare.^29,31,32,34,36,39^ The healthcare experience of some of these healthcare providers ranged from 1 to 42 y.^25,27^

### GDM screening, diagnosis and management practices

Detection and management practices for GDM varied substantially across healthcare facilities in Africa. Except for the studies conducted by Mensah et al., Njete et al. and Nielsen et al., who reported universal screening in some health facilities in Ghana, Tanzania and Cameroun,^27,30,37,39^ a selective screening approach dominated the majority of healthcare settings.^25,26,29,32,33,35,37,38^

Regarding diagnostic approaches, 2013 WHO diagnostic criteria were adopted by some facilities. However, pregnant women expressed concerns with the tolerability and acceptability of the test and shortage of diagnostic resources.^30,34,37^ In a study conducted by Nielsen et al. on compliance and acceptability of screening and diagnosing procedures, health professionals in Kenya raised concerns about the nauseating effect of the 75 g glucose load used for the OGTT. Hence they experimented with 300 ml of sprite (a non-alcoholic drink), which by comparison had a less nauseating effect.^37^ In terms of the gestational age for screening, while some health facilities screened pregnant women at 24–28 wk, others were screened at 16–34 wk.^35^ Three studies, from Morocco, Nigeria and South Africa, reported screening for GDM at the initiation of antenatal care and sometimes after the first trimester.^26,29,39^

In assessing management practices, two studies reported insulin and metformin as the medications of choice for managing GDM and emphasised dietary and lifestyle modification as an alternative to achieving glucose control.^26,39^ Beyond medical intervention, healthcare providers in South Africa mentioned comprehensive non-pharmacological interventions such as peer group teaching and group or individual counselling with a dietician or healthcare professional as effective GDM management practices.^26^

## Themes generated from the review

We present the findings in line with the review objectives: (1) barriers to screening and diagnosis, (2) hindrances to implementing management interventions and (3) the experiences of women regarding GDM diagnosis and management. Through the thematic content synthesis, we generated three themes that contextualised women's experiences regarding the continuum of GDM care overlapping the three objectives of the review. These three themes comprised health system, patient-related and sociocultural barriers limiting GDM screening, diagnosis and management. Essentially, most of the experiences stemmed from lack of empathy and inadequate interaction with health providers coupled with inadequate social support from family and friends.^26,27,30,31,35,37,38^

### Barriers to GDM screening and diagnosis

In Supplementary Table 1, we summarise the health system- and patient-related barriers to initiating screening and diagnostic strategies for GDM.

#### Health system-related barriers to GDM screening and diagnosis

Overall, seven studies reported barriers to screening and diagnosis of GDM.^29,30,33,34,37–39^ Two studies reported barriers from the perspective of pregnant women and women previously diagnosed with GDM,^30,35^ whereas the remaining five studies included views of GDM programme implementors, as well as health professionals, in addition to women diagnosed with GDM or DIP.^30,34,37–39^ A few of the studies reported on the shortage of trained health professionals as a barrier to GDM screening and diagnosis,^33,34,39^ which led to healthcare professionals' inability to comprehensively provide health education and counselling support throughout pregnancy.^25,27,29,30,32,33,36^ Beyond this, the few healthcare professionals at post do not have the requisite skills to provide GDM services.^31,33,34,38^ Some studies attributed the lack of requisite skills among professionals to the limited opportunities for in-service training on the GDM care process^33,34,38,39^ due to the emerging nature of guidelines on its management.

On the other hand, Muhwava et al. concluded that healthcare professionals do not satisfactorily explain GDM screening and diagnostic procedures.^33^ Often, healthcare providers are unable to follow up women after the first antenatal visit, even among those who test positive for glucosuria or are scheduled for subsequent testing.^30,34,39^ Although this may be due to the high patient turnout that characterises many antenatal clinics, it may be exacerbated by staff shortages, insufficient space or inadequate logistics and consumables.^34,37^ An absence of protocols and guidelines also hamper screening and diagnosis, especially among newly recruited health professionals who may not be acquainted with the GDM care regimen.^29,34,37^ Nwose et al. found non-adherence to GDM protocols and guidelines despite their availability in some health facilities.^29^ This could result in long waiting hours at antenatal clinics, which could deter pregnant women who travel long distances from returning for subsequent antenatal care services. Meanwhile, some pregnant women leave the antenatal clinics without undergoing the prescribed test, especially if they are required to fast overnight for 8 h before the test.^29^

#### Patient-related barriers to GDM screening and diagnosis

The intention to screen and diagnose GDM commences at the initiation of antenatal care. However, some women begin antenatal visits beyond the 24–28 wk period recommended for GDM testing.^29,34,37^ Also, pregnant women are unable to accurately tell their last menstrual date,^29^ while others tend to under-report their diabetes risk,^37^ which potentially affects the decision to test, especially in settings where a risk-based approach to GDM screening is practised. Njete et al. reported lost to follow-up among pregnant women as a significant barrier to GDM screening.^29,30^ In terms of acceptability of the diagnostic test, Nielsen et al. and Nwose et al. deduced that the glucose solution used for the OGTT and the 8-h prerequisite fasting are unbearable for some pregnant women.^29,35,37^

### Barriers to GDM management

Health system- and patient-related barriers adversely affecting the provision of management interventions for pregnant women diagnosed with GDM are summarised in Supplementary Table 2. In all instances, the gaps we identified were related to the management of GDM.

#### Health system-related barriers to the management of GDM

Seven studies sampled the experiences of health professionals, pregnant women and women previously diagnosed with GDM or DIP on barriers to the management of GDM.^25–27,32,34,36,39^ Four studies highlighted inadequate health professionals at various levels of care as a significant barrier to its management.^25,26,38^ Here, too, insufficient knowledge about GDM, its management practices, as well as inadequate training on relevant skills for GDM compared with HIV, malaria and TB in the subregion, were identified as significant barriers to GDM management.^25,26,33,34,38^ In a study conducted by Nielsen et al., women diagnosed with GDM cited inadequate knowledge of healthcare providers on menu planning as the reason why dietary compliance remains a challenge.^38^ Regarding health service delivery, poor coordination and communication lapses between health providers and pregnant women disrupt the continuity of GDM care and treatment adherence.^25,27,33,36,39^ Within the healthcare system, Nielsen et al. identified a weak referral system and difficulty accessing specialist care as barriers to GDM care.^39^

Besides human resources, the shortage of medications, glucose strips, glucometers and reagents also poses a significant challenge to GDM care.^25,34,38^ In two studies conducted by Mukona et al., healthcare providers mentioned the absence of diabetic medications as a hindrance to therapy compliance.^25,36^

#### Patient-related barriers to the management of GDM

Three studies identified financial constraints and the absence of insurance systems as significant barriers to GDM management. For example, in healthcare settings where the cost of maternal health services is not covered by any form of insurance, women within the lower wealth index are constrained in purchasing medications, glucose strips and following the prescribed nutritional guidelines for managing GDM.^27,32,36^ Also, difficulty in comprehending the treatment regimen and painful insulin injections hinder adherence to antidiabetic therapy.^32,36^

Beside medication, women with GDM cited family and societal support in GDM care. Some studies have linked the absence of significant support from family, peers and other social networks with poor treatment compliance and adverse outcomes.^27,32,36^ Patient support goes beyond family to healthcare providers with whom patients interact regularly. The need for treatment support from significant others emerged in two studies from Ghana, where women mentioned support from healthcare professionals and close relatives, particularly husbands, as a crucial component of GDM management.^27,31^ Utz et al., who assessed GDM screening and management practices in Morocco, documented a lack of empathy and understanding by healthcare providers as a significant setback to its management.^39^

### Sociocultural barriers

Three studies explored how finances, culture, customs and traditions influence GDM/DIP management. The cheaper cost of herbal medicine encouraged some diagnosed women to consult traditional healers. Others mentioned pressure from family and friends, who believe that GDM is caused by spiritual or mystical forces, as the reason for consulting herbalists.^25,32,36^

Furthermore, some cultures and religions forbid women from eating certain foods, even if they have positive implications for GDM treatment outcomes.^31^ Concerning societal barriers, Nielsen et al. reported the perceptions of women regarding their body size, particularly during pregnancy, as a barrier to GDM management.^38^ In typical rural settings, losing weight during pregnancy creates an impression of ill-health and poverty. In such settings, compliance with dietary guidelines that require optimum weight gain during pregnancy is often low.^38^

### Experiences of GDM women on detection and management

As summarised in Supplementary Table 3, women experience sadness, anxiety and mixed feelings while accessing GDM services, particularly before and after GDM diagnosis, largely due to the failure of health professionals to explain test procedures.^31,33^ The mixed feelings were attributed to inadequate interaction with health providers and a lack of reassurance concerning positive treatment outcome.^36^ Given the perceived spiritual connotation behind GDM, some women might keep the condition a secret for fear of stigmatisation.^31^ Few authors have indicated the need to prioritise the psychological well-being of women diagnosed through counselling and health education.^27,31^

## Discussion

This review highlights barriers to screening, diagnosis and management of GDM and experiences of women with GDM in Africa. Perspectives obtained from healthcare providers and patients reveal barriers to the detection and management of GDM within the health system. Other key barriers included sociocultural and religious dimensions that affect health-seeking behaviour. Generally, the barriers are consistent across the studies included in this review, except for sociocultural barriers, which differed according to the country context.

Although the findings of this review cannot be universally adjudged, it establishes systematic gaps and inadequate attention to GDM, which constitutes one of the most significant burdens to diabetes in Africa.^2^ The foremost barrier to GDM detection and management is inadequate knowledge. Awareness of the condition among both healthcare providers and pregnant women will limit progression to diabetes type II. Other reviews have established evidence that a shortage of healthcare providers hinders GDM detection and management. However, the problem extends to a lack of knowledge resulting from limited opportunities for skills-based training.^40^ Because the majority of health services are concentrated at primary levels, this review proposes the need for capacity building at lower levels of care, alongside providing the essential equipment and consumables necessary to enhance GDM care.

Prioritising other diseases and programmes such as the prevention of mother-to-child transmission of HIV and intermittent preventive treatment of malaria at the expense of GDM is a concern. These programmes are prioritised and provide health professionals with an opportunity for training, but the same cannot be said of NCDs in pregnancy. While efforts have been made to improve NCD surveillance and response in LMICs, such interventions need to start from antenatal care clinics, where screening and diagnostic services start.

The multidimensional nature of the problems associated with GDM service provision require a comprehensive systemic revision to improve detection and management practices. As seen in this review, even in settings where protocols and guidelines exist, the feasibility of implementing WHO-recommended universal screening is problematic due to the scarcity of resources that characterises many healthcare settings in Africa. Therefore, there is a need for context-applicable screening and diagnostic protocols that are informed by cost, tolerability, availability, accessibility and sustainability to increase the uptake of GDM services.^17,37^ Otherwise, several pregnant women may go unscreened, to the detriment of the mother and baby. The few who test positive and require further testing or treatment are often not followed up or are lost to follow-up. Poor coordination of referral pathways is a significant factor given that most of the cases are managed at tertiary levels, which are grossly inaccessible to the majority of pregnant women. Inadequate contact and interaction with healthcare providers reduce chances for health education and counselling, which limit the effectiveness and satisfaction with care received. Moreover, patient education and counselling^41,42^ has been associated with positive treatment outcomes and facilitates postpartum care, which is key to early detection of type II diabetes.^43^ As antenatal care offers an opportunity for screening, diagnosing and management, healthcare providers should leverage this opportunity to enforce behavioural change through evidence-based interventions such as peer counselling and dietician consultation.

Cultural diversity within the African context is more pronounced, and management of GDM is not spared its consequence. Although several interventional studies have established the importance of dietary adherence for GDM management,^44,45^ lifestyle modification is disadvantaged by negative perceptions about weight gain during pregnancy and taboos and customs surrounding foods, thereby affecting adherence to the therapeutic regimen. Even in low-resource settings in developed countries including the UK, it is recommended that health education on diabetes in pregnancy incorporates culturally appropriate messages, as there are potential cultural issues, such as language and myths or ‘old wives tales’ that affect GDM treatment compliance.^46^

### Strength and limitations

This paper is the first review on barriers to GDM care in Africa and shows how GDM has received little attention in Africa. However, because of the growing interest in GDM research globally after the Hyperglycemia and Adverse Pregnancy Outcomes study in 2008, which reported adverse pregnancy outcomes per unit increase in maternal glucose,^47^ we observed that the eligible studies included in this review were published recently (during 2012–2019). The eligible studies included in the review represent the five subregions and different ethnic groups in Africa. More importantly, the included articles cut across qualitative, quantitative and mixed-method designs, which complementarily provide a contextual understanding of the challenges to GDM care. A considerable number of studies sampled diverse stakeholders concerned with GDM, thereby allowing a comprehensive description of the barriers to GDM service provision. All these elements enhanced the validity and generalisability of the findings across Africa.

Nevertheless, this review has some limitations. Generally, there was missing information on the gestational age of pregnant women at screening and the context of healthcare from which participants were recruited. The cultural and religious barriers identified may be unique to specific ethnic or religious groups and could be misleading if generalised because they vary from country to country and from one ethnic group to another, even in the same country. Nevertheless, they provide an insight that some barriers may be culturally inclined.

### Conclusions

This review shows the multidimensional factors that interact at different health systems and societal levels to hinder the detection and management of GDM in Africa. Insufficient clinical logistics, inadequate coordination of GDM care, limited human resources capacity and funding deficits grossly affect the testing and management of GDM in Africa. Women diagnosed experience anxiety, sadness, stigmatisation and uncertainty regarding treatment outcomes. Family support, customs and taboos are pertinent at the societal level. Broader consultation with key stakeholders to address these multifactorial challenges is essential in improving maternal and child health. The coexistence of infectious diseases continues to direct training needs, resource allocation and prioritisation of interventions. Nonetheless, pregnancy complications associated with GDM and its linkage with other NCDs is well established. Therefore, addressing these barriers is key to improving maternal and neonatal outcomes and promoting NCD-prevention strategies in Africa.

## Supplementary Material

ihab054_Supplemental_File

## Data Availability

None.
