# Supplementary material for: Barriers to screening, diagnosis and management of hyperglycaemia in pregnancy in Africa: a systematic review
Source: Int Health. 2021 Aug 25;14(3):211–21. doi: 10.1093/inthealth/ihab054 (PMC9070469; doi:10.1093/inthealth/ihab054)
Supplement: ihab054_Supplemental_File [file ihab054_supplemental_file.zip › Suplementary Data 1.docx]

**Results and search terms of databases accessed**

**Table 1: Name of Database: CINAHL**

PLATFORM: EBSCOhost

**Date of Search: 10/05/2020**

**Results: 22**

**Search terms:**

| Search id | Search terms | Results |
| --- | --- | --- |
| S1 | Barrier* | 96,932 |
| S2 | Challenge* | 197,931 |
| S3 | Obstacle* | 13,195 |
| S4 | Issue* | 349,168 |
| S5 | Experience* | 467,478 |
| S6 | Perception* | 169,242 |
| S7 | Attitude* | 365,433 |
| **S8** | **(S1 OR S2 OR S3 OR S4 OR S5 OR S6 OR S7)** | **1,299,544** |
| S9 | screening | 177,541 |
| S10 | detection | 101,590 |
| S11 | diagnosis | 920,333 |
| S12 | assessment | 722,578 |
| S13 | testing | 154,153 |
| S14 | Diagnostic test | 66,788 |
| S15 | management | 535,831 |
| S16 | Treatment | 1,137,538 |
| S17 | Diagnostic test (^[[1]](#footnote-1)^SU) | 4,560 |
| **S18** | **S9 OR S10 OR S11 OR S12 OR S13 OR S14 OR S15 OR S16 OR S17** | **2,706,260** |
| S19 | SU Diabetes Mellitus, Gestational | 7,424 |
| S20 | (^[[2]](#footnote-2)^MH "Diabetes Mellitus, Gestational") | 7,188 |
| S21 | (MH "Pregnancy in Diabetes+") | 9, 301 |
| S22 | hyperglycemia in pregnancy | 971 |
| S23 | diabetes in pregnancy | 13, 735 |
| S24 | GDM | 6,121 |
| S25 | Pregnancy-induced diabetes | 5,845 |
| S26 | Gestational diabetes mellitus | 8,982 |
| **S27** | **S19 OR S20 OR S21 OR S22 OR S23 OR S24 OR S25 OR S26** | **15,488** |
| S28 | Africa | 47,880 |
| S29 | {Algeria OR Angola…, OR Zimbabwe} | 95,237 |
| S30 | **S28 OR S29** | **107,148** |
| S31 | S8 AND S18 AND S27 AND S30 | 22 |

**Table 2: Name of Database: PubMed:**

PLATFORM: PUBMED

**Date of Search: 08-09/05/2020**

**Results:112**

| Search ID | Search Term | Results |
| --- | --- | --- |
| #1 | Barrier* | 305,909 |
| #2 | Challenge* | 642,712 |
| #3 | Obstacle* | 47,614 |
| #4 | Issue* | 568, 062 |
| #5 | Experience* | 1,057,067 |
| #6 | Perception* | 433,699 |
| #7 | Attitude* | 418,945 |
| **#8** | **((((((#1) OR (#2)) OR (#3)) OR (#4)) OR (#5)) OR (#6)) OR (#7)** | **2,987,999** |
| #9 | screening | 4,918,714 |
| #10 | detection | 2,298,163 |
| #11 | diagnosis | 10,085,068 |
| #12 | assessment | 3,253,705 |
| #13 | testing | 2,765,095 |
| #14 | diagnostic test (^[[3]](#footnote-3)^MeSH Term) | 12, 005 |
| #15 | diagnostic test | 191,119 |
| #16 | treatment | 11,144,142 |
| #17 | management | 3,002,269 |
| **#18** | **((((((((#9) OR (#10)) OR (#11)) OR (#12)) OR (#13)) OR (#14)) OR (#15)) OR (#16)) OR (#17)** | **19,822,047** |
| #19 | Diabetes, Gestational (MeSH Terms) | 12,364 |
| #20 | Diabetes, Pregnancy-Induced | 1,302 |
| #21 | Diabetes, Pregnancy Induced | 23,990 |
| #22 | gestational diabetes mellitus | 21, 976 |
| #23 | Hyperglycaemia in pregnancy | 4,034 |
| #24 | Diabetes in pregnancy | 41,302 |
| #25 | GDM | 7, 309 |
| #26 | Diabetes in pregnanc* | 40, 180 |
| **#27** | **(((((((#19) OR (#20)) OR (#21)) OR (#22)) OR (#23)) OR (#24)) OR (#25)) OR (#26)** | **43,559** |
| #28 | Africa | 360, 669 |
| #29 | {Algeria OR Angola…, OR Zimbabwe} | 686,411 |
| **#30** | **(#28) OR (#29)** | **725,729** |
| **#31** | **(((#8) AND (#18)) AND (#27)) AND (#30)** | **112** |
|  |  |  |

**Table 3: Name of Database: APA PSYCHINFO**

PLATFORM: EBSCOhost

**Date of Search: 09/05/2020**

**Results: 5**

**Search terms:**

| **Search ID** | **Terms** | **Results** |
| --- | --- | --- |
| S1 | Barrier* | 73,302 |
| S2 | Challenge* | 206,002 |
| S3 | Issue* | 615,321 |
| S4 | Obstacle* | 19,169 |
| S5 | Experience* | 666,894 |
| S6 | Perception* | 558,615 |
| S7 | Attitude* | 571,332 |
| **S8** | **S1 OR S2 OR S3 OR S4 OR S5 OR S6 OR S7** | **2,031,235** |
| S9 | screening | 84, 029 |
| S10 | detection | 66, 123 |
| S11 | diagnosis | 231,848 |
| S12 | assessment | 516, 879 |
| S13 | testing | 244,285 |
| S14 | Diagnostic test | 44,501 |
| S15 | SU Diagnostic test | 7,240 |
| S16 | management | 361,133 |
| S17 | Treatment | 895,973 |
| **S18** | **S8 OR S9 OR S10 OR S11 OR S12 OR S13 OR S14 OR S15 OR S17** | **1,800,559** |
| S19 | Gestational diabetes | 695 |
| S20 | SU Gestational diabetes | 369 |
| S21 | diabetes in pregnancy | 1,063 |
| S22 | Hyperglycemia in pregnancy | 49 |
| S23 | Pregnancy-induced diabetes | 29 |
| S24 | ^[[4]](#footnote-4)^GDM | 327 |
| S25 | **S19 OR S20 OR S21 OR S22 OR S23 OR S24** | 1,317 |
| S26 | Africa | 35,544 |
| S27 | {Algeria OR Angola…, OR Zimbabwe} | 68,574 |
| **S28** | **S26 OR S27** | **73,567** |
| S25 | **S8 AND S18 AND S25 AND S28** | 5 |

**Table 4: Name of Database: Web of Science**

PLATFORM: Web of Science Collection

**Date of Search: 10/05/2020**

**Results: 95**

**Search terms:**

| set | Search term | Results |
| --- | --- | --- |
| #1 | ALL^[[5]](#footnote-5)^=(Barrier*) | 567,455 |
| #2 | ALL=(Challenge*) | 989,158 |
| #3 | ALL=(Issue*) | 1,103,119 |
| #4 | ALL=(Experience*) | 1,422,896 |
| # 5 | ALL=(Obstacle*) | 87, 224 |
| #6 | ALL=(Perception*) | 520,634 |
| #7 | ALL=(Attitude*) | 298, 133 |
| **# 8** | **#1 OR #2 OR #3 OR #4 OR #5 OR #6 OR #7** | **4,353,396** |
| #9 | ALL=(screening) | 853,547 |
| # 10 | ALL=(diagnosis) | 1,511,906 |
| #11 | ALL=(diagnostic test) | 189,424 |
| #12 | ALL=(testing) | 4,379,853 |
| #13 | ALL=(management) | 2,755,025 |
| #14 | ALL=(treatment) | 4,650,957 |
| #15 | ALL=(assessment) | 1,531,553 |
| #16 | ALL=(detection) | 1,376,411 |
| **#17** | **#16 OR #15 OR #14 OR #13 OR #12 OR #11 OR #10 OR #9** | **13,231,884** |
| #18 | ALL=GDM | 8,372 |
| #19 | ALL=(Gestational Diabetes Mellitus) | 12,741 |
| #20 | ALL=(Hyperglycemia in Pregnancy) | 2,394 |
| #21 | ALL=(Diabetes in pregnancy) | 24,280 |
| #22 | ALL=(pregnancy-induced diabetes) | 804 |
| #23 | ALL=(Gestational diabetes) | 21,158 |
| #24 | TS=(gestational diabetes) | 19,668 |
| **#25** | **#26 OR #25 OR #24 OR #23 OR #22 OR #21 OR #20 #19** | **34,731** |
| **#**26 | ALL=(Africa) | **543,327** |
| #27 | ALL ={Algeria OR ALL=Angola…,ALL=Zimbabwe} | 1,415,182 |
| **#28** | **#26 OR #27** | **1,496,337** |
| **#29** | **#28 AND #25 AND #17 AND #8** | **95** |

**Table 5: Name of Database: WHOLIS:** PLATFORM: Global Health Library

**Date of Search: 09/05/2020**

**Results: 14**

**Search terms:**

| # | Search string | # results |
| --- | --- | --- |
| 1 | ^[[6]](#footnote-6)^tw:("barrier" OR "experience" OR "issues" OR "obstacle" OR "Perception" OR "Attitude" OR "Challenge") | 2,290,756 |
| 2 | tw:("diagnosis" OR "diagnostic test" OR "testing" OR "screening" OR "treatment" OR "management" OR "assessment") | 11,682,119 |
| 3 | tw:("gestational diabetes" OR "gdm" OR "diabetes in pregnancy" OR "Diabetes, Gestational" OR "hyperglycaemia in pregnancy" OR "Pregnancy in Diabetics") | 26,823 |
| 4 | (tw:("Algeria" OR "Angola" OR "Benin" OR "Botswana" OR "Burkina Faso" OR "Burundi" OR "Cameroon" OR "Cape Verde" OR "Central African" OR "Republic Chad" OR "Comoros" OR "Congo-Brazzaville" OR "Congo-Kinshasa" OR "Cote d'Ivoire" OR "Djibouti" OR "Egypt" OR "Equatorial" OR "Guinea" OR "Eritrea" OR "Ethiopia" OR "Gabon" OR "Gambia" OR "Ghana" OR "Guinea"or "Guinea Bissau" OR "Kenya" OR "Lesotho" OR "Liberia" OR "Libya" OR "Madagascar" OR "Malawi" OR "Mali" OR "Mauritania" OR "Mauritius" OR "Morocco" OR "Mozambique" OR "Namibia" OR "Niger" OR "Nigeria" OR "Rwanda" OR "Senegal" OR "Seychelles" OR "Sierra Leone" OR "Somalia" OR "South Africa" OR "South Sudan" OR "Sudan" OR "Swaziland" OR "São Tomé Príncipe" OR "Tanzania" OR "Togo" OR "Tunisia" OR "Uganda" OR "Western Sahara" OR "Zambia" OR "Zimbabwe")) | 9,137,173 |
| 5 | (tw:("Africa")) | 208,598 |
| 6 | 1 AND 2 AND 3 AND 4 AND 5 | 14 |

**Appendix 1: Search strings for the various databases**

PUBMED

Search strategy: (((Barrier*) OR (Challenge*) OR (Obstacle*) OR (Issue*) OR (Experience*) OR (Perception*) OR (Attitude*)) AND ((screening) OR (detection) OR (diagnosis) OR (assessment) OR (testing) OR (diagnostic test[MeSH Terms]) OR (diagnostic test) OR (Treatment) OR (management)) AND ((gestational diabetes[MeSH Terms]) OR (gestational diabetes) OR (Diabetes, Pregnancy-Induced) OR (Diabetes, Pregnancy Induced) OR (gestational diabetes mellitus) OR (Hyperglycaemia in pregnancy) OR (Diabetes in pregnancy) OR (GDM) OR (diabetes in pregnanc*)) AND ((Africa) OR (Algeria OR Angola OR Benin OR Botswana OR (Burkina AND Faso) OR Burundi OR Cameroon OR (Cape AND Verde) OR (Central AND African) OR (Republic AND Chad) OR Comoros OR Congo-Brazzaville OR Congo-Kinshasa OR (Cote AND d'Ivoire) OR Djibouti OR Egypt OR Equatorial OR Guinea OR Eritrea OR Ethiopia OR Gabon OR Gambia OR Ghana OR Guinea OR (Guinea AND Bissau) OR Kenya OR Lesotho OR Liberia OR Libya OR Madagascar OR Malawi OR Mali OR Mauritania OR Mauritius OR Morocco OR Mozambique OR Namibia OR Niger OR Nigeria OR Rwanda OR Senegal OR Seychelles OR (Sierra AND Leone) OR Somalia OR (South AND Africa) OR (South AND Sudan) OR Sudan OR Swaziland OR (São AND Tomé AND Príncipe) OR Tanzania OR Togo OR Tunisia OR Uganda OR (Western AND Sahara) OR Zambia OR Zimbabwe)))

**CINAHL**

(barrier* OR challenge* OR issue* OR obstacle* OR experience*) AND (screening OR detection OR assessment OR testing OR ((diagnostic AND test)) OR treatment OR management) AND (((gestational AND diabetes)) OR ((diabetes AND "in" AND pregnancy)) OR GDM OR ((gestational AND diabetes AND mellitus)) OR (DE "Gestational Diabetes" OR DE "Hyperglycemia")) AND (Africa OR ALL=(Algeria OR Angola OR Benin OR Botswana OR (Burkina AND Faso) OR Burundi OR Cameroon OR (Cape AND Verde) OR (Central AND African) OR (Republic AND Chad) OR Comoros OR Congo-Brazzaville OR Congo-Kinshasa OR (Cote AND d'Ivoire) OR Djibouti OR Egypt OR Equatorial OR Guinea OR Eritrea OR Ethiopia OR Gabon OR Gambia OR Ghana OR Guinea OR (Guinea AND Bissau) OR Kenya OR Lesotho OR Liberia OR Libya OR Madagascar OR Malawi OR Mali OR Mauritania OR Mauritius OR Morocco OR Mozambique OR Namibia OR Niger OR Nigeria OR Rwanda OR Senegal OR Seychelles OR (Sierra AND Leone) OR Somalia OR (South AND Africa) OR (South AND Sudan) OR Sudan OR Swaziland OR (São AND Tomé AND Príncipe) OR Tanzania OR Togo OR Tunisia OR Uganda OR (Western AND Sahara) OR Zambia OR Zimbabwe))

**WHOLIS**

(tw:("barrier" OR "experience" OR "issues" OR "obstacle" OR "Perception" OR "Attitude" OR "Challenge")) AND (tw:("diagnosis" OR "diagnostic test" OR "testing" OR "screening" OR "treatment" OR "management" OR "assessment")) AND (tw:("gestational diabetes" OR "GDM" OR "diabetes in pregnancy" OR "Diabetes, Gestational" OR "hyperglycaemia in pregnancy" OR "Pregnancy in Diabetics")) AND (tw:("Algeria" OR "Angola" OR "Benin" OR "Botswana" OR "Burkina Faso" OR "Burundi" OR "Cameroon" OR "Cape Verde" OR "Central African" OR "Republic Chad" OR "Comoros" OR "Congo-Brazzaville" OR "Congo-Kinshasa" OR "Cote d'Ivoire" OR "Djibouti" OR "Egypt" OR "Equatorial" OR "Guinea" OR "Eritrea" OR "Ethiopia" OR "Gabon" OR "Gambia" OR "Ghana" OR "Guinea"or "Guinea Bissau" OR "Kenya" OR "Lesotho" OR "Liberia" OR "Libya" OR "Madagascar" OR "Malawi" OR "Mali" OR "Mauritania" OR "Mauritius" OR "Morocco" OR "Mozambique" OR "Namibia" OR "Niger" OR "Nigeria" OR "Rwanda" OR "Senegal" OR "Seychelles" OR "Sierra Leone" OR "Somalia" OR "South Africa" OR "South Sudan" OR "Sudan" OR "Swaziland" OR "São Tomé Príncipe" OR "Tanzania" OR "Togo" OR "Tunisia" OR "Uganda" OR "Western Sahara" OR "Zambia" OR "Zimbabwe")) AND (tw:("Africa"))

**PSYCHINFO**

(barrier* OR challenge* OR issue* OR obstacle* OR experience*) AND (screening OR detection OR assessment OR testing OR ((diagnostic AND test)) OR treatment OR management) AND (((gestational AND diabetes)) OR ((diabetes AND "in" AND pregnancy)) OR GDM OR ((gestational AND diabetes AND mellitus)) OR (DE "Gestational Diabetes" OR DE "Hyperglycemia")) AND (Africa OR (Algeria OR Angola OR Benin OR Botswana OR (Burkina AND Faso) OR Burundi OR Cameroon OR (Cape AND Verde) OR (Central AND African) OR (Republic AND Chad) OR Comoros OR Congo-Brazzaville OR Congo-Kinshasa OR (Cote AND d'Ivoire) OR Djibouti OR Egypt OR Equatorial OR Guinea OR Eritrea OR Ethiopia OR Gabon OR Gambia OR Ghana OR Guinea OR (Guinea AND Bissau) OR Kenya OR Lesotho OR Liberia OR Libya OR Madagascar OR Malawi OR Mali OR Mauritania OR Mauritius OR Morocco OR Mozambique OR Namibia OR Niger OR Nigeria OR Rwanda OR Senegal OR Seychelles OR (Sierra AND Leone) OR Somalia OR (South AND Africa) OR (South AND Sudan) OR Sudan OR Swaziland OR (São AND Tomé AND Príncipe) OR Tanzania OR Togo OR Tunisia OR Uganda OR (Western AND Sahara) OR Zambia OR Zimbabwe))

**Web of Science**

ALL=(Algeria) OR ALL=(Angola) OR ALL=(Benin) OR ALL=(Botswana) OR ALL=(Burkina Faso) OR ALL=(Burundi) OR ALL=(Cameroon )OR ALL=(Cape Verde) OR ALL=(Central African) OR ALL=(Republic Chad) OR ALL=(Comoros) OR ALL=(Congo-Brazzaville) OR ALL=(Congo-Kinshasa) OR ALL=(Cote d'Ivoire) OR ALL=(Djibouti) OR ALL=(Egypt) OR ALL=(Equatorial) OR ALL=(Guinea) OR ALL=(Eritrea) OR ALL=(Ethiopia) OR ALL=(Gabon) OR ALL=(Gambia) OR ALL=(Ghana) OR ALL=(Guinea) OR ALL=(Guinea Bissau) OR ALL= (Kenya) OR ALL=(Lesotho) OR ALL=(Liberia) OR ALL=(Libya) OR ALL=(Madagascar) OR ALL=(Malawi) OR ALL=(Mali) OR ALL=(Mauritania) OR ALL=(Mauritius) OR ALL=(Morocco) OR ALL=(Mozambique) OR ALL=(Namibia) OR ALL=(Niger) OR ALL=(Nigeria) OR ALL=(Rwanda) OR ALL=(Senegal) OR ALL=(Seychelles) OR ALL=(Sierra Leone) OR ALL=(Somalia) OR ALL=(South Africa) OR ALL=(South Sudan) OR ALL=(Sudan) OR ALL=(Swaziland) OR ALL=(São Tomé Príncipe) OR ALL=(Tanzania) OR ALL=(Togo) OR ALL=(Tunisia) OR ALL=(Uganda) OR ALL=(Western Sahara) OR ALL=(Zambia) OR ALL= (Zimbabwe)

**Google Scholar**

(Gestational diabetes OR diabetes in pregnancy OR hyperglycemia in pregnancy OR GDM OR pregnancy-Induced Diabetes) AND (barrier* OR challenge* OR experience* OR Issue* OR Perception*) AND (screening OR diagnosis OR management) AND (Africa)

1. SU=Major Subject and Minor Subjects [↑](#footnote-ref-1)
2. MH=Exact Subject Heading [↑](#footnote-ref-2)
3. MeSH=Medical Subject Heading [↑](#footnote-ref-3)
4. GDM=Gestational diabetes mellitus [↑](#footnote-ref-4)
5. ALL=All feilds [↑](#footnote-ref-5)
6. Tw=Text word [↑](#footnote-ref-6)
