# Supplementary material for: Barriers to screening, diagnosis and management of hyperglycaemia in pregnancy in Africa: a systematic review
Source: Int Health. 2021 Aug 25;14(3):211–21. doi: 10.1093/inthealth/ihab054 (PMC9070469; doi:10.1093/inthealth/ihab054)
Supplement: ihab054_Supplemental_File [file ihab054_supplemental_file.zip › Supplementary Table 1.docx]

*Supplementary Table 1:* *Summary of findings on barriers to screening and diagnosis of GDM (Alphabetical order, n=7)*

| Authors/year/  Country | Aim of the study | Key findings | Main gaps and recommendations |
| --- | --- | --- | --- |
| *(Nwose* et al.*, 2019)* ^(29)^  Nigeria | To assess barriers to  GDM diagnosis and postpartum follow-up; to determine educational needs | ***Health system barriers***   - Screening not done routinely, - Limited resources for screening and diagnosis, - Overcrowding at antenatal clinics, - Loss to follow-up   ***Patient-related barriers***   - Failure of pregnant women to adhere to appointment schedules - Inability to tell last menstrual date - Late registration of antenatal by pregnant women. - Long-distance to antenatal clinics - Transportation issues to health centers. | Health systems need to be re-oriented to address issues of resources to meet the health educational needs of pregnant women. |
| (Njete *et al.*, 2018) ^(30)^  Tanzania | To determine the prevalence and predictors of GDM as well as acceptability of pregnant women to return for glucose tolerance testing among | ***Health system barriers***   - Loss of follow up on women eligible for OGTT and FBG test.   ***Patient-related barriers***   - Low socioeconomic status - Low level of education affected return for further test | Improving funding for GDM services and comprehensive health education can be helpful. |
| (Nielsen, de Courten and Kapur, 2012a) ^(37)^  Sudan, Kenya, Cameroun and other LMICs^a^ | Investigate whether WDF GDM projects utilize guidelines, its applicability and usefulness for detecting GDM, and barriers in implementing such guidelines | ***Patient related barriers***   - Late registration of antenatal by pregnant women - Women under-report their risk for DM, which is relevant for initiation for screening - Test consumes time, nausea associated with drinking glucose solution - Lack of consumables. | Beyond logistics, late initiation of ANC, and side effect of test solutions also affect GDM detection. Pre-test counselling must focus on mitigating these factors to secure patient compliance. |
| *(Ugboma, Aburoma and Ukaigwe, 2012)* ^(35)^  Nigeria | To identify the incidence of undiagnosed GDM and its consequences on maternal/neonatal mortality and importance of screening | ***Patient related barriers***   - Declining glucose challenge test as pregnant women could not fast overnight - Fear of invasive procedures - Concern over effect on unborn baby - Ignorance among some women about effect of GDM on the unborn baby. | There is a perception of danger of test procedures to both mother and the unborn child. Even though there is evidence of the benefit of universal screening, the concerns of pregnant women need to be resolved during the initiation of antenatal care to keep them informed and anxiety allayed. |
| (Utz et al., 2016) ^(39)^  Morocco | To provide a comprehensive picture of the current situation on screening and management of GDM at different levels of care and to highlight existing challenges. | ***Health system barriers***   - Low sensitization on GDM - Women did not receive information about GDM Women had no opportunity to ask question during ANC - Long travel distance to get tested - Some pregnant women may not be fasting by the time they get to the laboratory - Poor communication between healthcare providers and patients. - GDM management occurs at tertiary facilities rather than primary level healthcare | Poor arrangement of health services hinders delivery of GDM services. Addressing these concerns require building the capacity of primary level healthcare workers to diagnose and manage GDM. |
| (Woticha, Deressa and Reja, 2019) ^(34)^  Ethiopia | To explore barriers towards detection and management of GDM | ***Health system barriers***   - Lack of awareness by healthcare providers on GDM - Lack of standard guidelines and protocols - Inadequately trained healthcare providers - Frequent shortages of logistics and supplies for GDM screening.   ***Patient related barriers***   - Mothers refusing to attend ANC early enough to allow initiation of GDM screening and diagnosis. | Aside logistical constraints, critical health professionals and lack of training opportunities affect GDM detection. Managers must aim to expand the numbers and build capacity of providers through in-service training. |
| (Nielsen et al., 2012b) ^(38)^  Sudan, Kenya, Cameroon and other LMICs ^b^ | To identify health system and societal barriers hindering detection and treatment of GDM | ***Health system barriers***   - Lack of trained health care providers - especially female doctors - High staff turnover - Lack of awareness among health professionals - Lack of GDM knowledge among healthcare - Lack of standard protocols on GDM care - Poor transportation network - Weak referral systems and poor follow up systems Absence of consumables and test kits.   ***Societal barriers***   - Perception of female body size which prevent pregnant women from complying with dietary advice. | Healthcare workers need to emphasis the benefit of weight control during pregnancy through durbars and other educational platforms. Such educations must aim at addressing myths and misconceptions surrounding GDM |

**Abbreviations: OGTT, Oral glucose tolerance test, GDM, gestational diabetes mellitus, DIP, diabetes in pregnancy, FBG, fasting blood glucose, LMICs low middle-income countries,**

**^a^Other LMICs, India, Cuba, China.**

**^b^India, Cuba, Jamaica, China.**

**NB, Nielsen et al., 2012b and Woticha et al., provided multiple data about two objectives of the review and hence, their findings were categorized and repeated Supplementary Data 1 and 2**
