# Supplementary material for: Barriers to screening, diagnosis and management of hyperglycaemia in pregnancy in Africa: a systematic review
Source: Int Health. 2021 Aug 25;14(3):211–21. doi: 10.1093/inthealth/ihab054 (PMC9070469; doi:10.1093/inthealth/ihab054)
Supplement: ihab054_Supplemental_File [file ihab054_supplemental_file.zip › Supplementary Table 2.docx]

Supplementary Table 2: Summary of findings on barriers to management of GDM (Alphabetical order, n=8)

| Authors/year/  Country | Aim of the study | Key findings | | Main gaps and recommendations | |
| --- | --- | --- | --- | --- | --- |
| (Mukona et al., 2017a) ^(32)^  Zimbabwe | To explore the barriers to adherence and possible solutions to non-adherence to antidiabetic therapy in women with diabetes in pregnancy. | ***Health system barrier***   - Lack of finances - Lack of health education on DIP - Lack of trained health providers - Poor coordination among health workers - Shortage of relevant staff   ***Facilitators of GDM care***   - Subsidization of care - Improving health education - Formal training of health workers - Improving coordination among health professionals - Dedicating an established unit for DIP women | | While providing logistical support and capacity building for relevant health providers forms the base of GDM care, reorientation of GDM services and some financial support will benefit detection and management practices | |
| (Mukona et al., 2017b) ^(25)^  Zimbabwe | Patients’ perspective on barriers and solutions to adherence in anti-diabetic therapy in pregnancy | ***Patient-related barriers***   - Low socioeconomic status of women - High cost of orthodox medicine - Lack of support, effects of pregnancy - Complicated treatment regimen - Weak health system - Complicated DIP disease process.   ***Socio-cultural factors***   - Belief that GDM is caused by demons and other spiritual forces   ***Facilitators of GDM care***   - Strong support from the community - Financial support - Improvement in GDM services. | | Treatment plan that includes invasive procedures for pregnant women affect GDM management and leads to defaulting and poor compliance. Providing adequate support and addressing cultural barriers through treatment support groups could be of benefit. | |
| *(Muhwava* et al.*, 2019)* ^(33)^  South Africa | To explore lived experiences of women’s with GDM and the feasibility of  sustained lifestyle modification after GDM | ***Health system barriers***   - Health workers not having time to explain GDM diagnosis - Lack of counselling and inadequate logistics and materials for GDM management.   ***Patient related barriers***   - Limited understanding of GDM - Emotional effects on diagnosis - Physical discomfort during pregnancy - Influence of the environment dominated with unhealthy foods.   ***Facilitators of GDM care***   - Affordability of foo - Social support and social norms. - ***Motivation for lifestyle changes*** - Concern for healthy baby - Concern about own health - Emotional support during diagnosis | | Lifestyle modification is essential in GDM care and requires measures such as effective counselling and updated knowledge of health providers to support pregnant women and create an enabling healthcare environment to facilitate behavior changes among GDM women | |
| *(Muhwava* et al.*, 2018)* ^(26)^  South Africa | To explore policies and clinical practices on antenatal and post-natal care for GDM women and  stakeholders’ perspectives on barriers to and opportunities for delivering an integrated  maternal health service. | ***Health system barrier***s   - little emphasis on physical activity - Shortage of dieticians - Inadequate counselling on lifestyle changes - Affordability of healthy foods - Non-update of education materials - High patient volume - Socioeconomic challenges   ***Facilitators of GDM care***   - Concern for the health of the baby | | Creating an opportunity for training existing professionals might be helpful in filling the gap for specialized care for GDM services | |
| (Mukona *et al*., 2017c) ^(36)^  Zimbabwe | To develop an adherence promotion framework for  diabetes in pregnancy | ***Health system barriers***   - Financial constraints - Lack of health education on DIP - Lack of trained personnel - Weak collaboration and shortage of staff,   ***Patient-related barriers***   - Low socioeconomic status of pregnant women - Lack of support from family and friends - Complications in pregnancy - Complicated treatment regimen - Poor service at the hospital - Seeking cure from faith healers.   ***Facilitators***   - **S**ubsidization of healthcare - Strengthening health education - Formal training of healthcare worker - Effective collaboration among health workers Concern for healthy baby - Family and peer support - Financial support, - Improving quality of healthcare | | The study highlights barriers across the continuum of GDM care from health systems to patient and the society. A framework for GDM care will improve healthcare worker capacity and leverage support from the family and community to address challenges with GDM care particularly taboos and customs that affect compliance with GDM care | |
| (Woticha, Deressa and Reja, 2019) ^(34)^  Ethiopia | To explore barriers to the detection and management of GDM | ***Health system barriers***   - Lack of awareness of GDM among healthcare providers - Lack of standard guidelines and protocols Inadequate training of providers - Frequent shortages of logistics and supplies for GDM screening.   ***Patient Related barriers***   - Late ANC registration - Lack of awareness on GDM | Aside logistic constraints, lack of critical health professionals and training affect GDM detection. Healthcare managers must aim at improving numbers of providers and build their capacity through in-service training. | |  |
| (Mensah *et al*., 2019) ^(27)^  Ghana | Describe the experiences of women regarding the nursing management they received after GDM diagnosis; and the perceptions of nurse-midwives on nursing management of GDM | ***Health system related barriers***   - Inadequate health education by health professionals - Lack of awareness among health professionals and GDM women   ***Patient barriers***   - Financial constraints that challenges clinic attendance - Inability to buy healthy foods - Shortage Insulin, glucose strips and lack of storage for insulin at home   ***Societal barriers***   - Taboos on some diets which are nutritious for pregnant women - Inadequate support from family, peer and community | The study highlights the systemic, patient and societal level barriers to GDM management and the need for a comprehensive approach to address these key challenges. | |  |
| (Nielsen et al., 2012b) ^(38)*^  Sudan, Kenya, Cameroon and other LMICs^b^ | Investigate whether WDF GDM projects utilize guidelines, its applicability and usefulness for screening  and diagnosis of GDM, and barriers in implementing such guidelines | ***Health system barriers***   - Lack of trained health care providers - especially female doctors - High staff turnover - lack awareness among health professionals, lack of knowledge among healthcare, - lack of standard protocols on GDM care - Poor transportation - Weak referral systems and poor follow up systems - Absence of consumables and test kits.   ***Societal barriers***   - Identified were perception of female body size which prevent pregnant women from complying with dietary advice. | Health care workers need to emphasis the benefit of appropriate pregnancy weight gain measures through durbars and other educational platforms. ^[[1]](#footnote-1)^ | |  |

**Abbreviations: OGTT, Oral glucose tolerance test, GDM, gestational diabetes mellitus, DIP, diabetes in pregnancy, FBG, fasting blood glucose.**

**NB, Nielsen et al., 2012b and Woticha et al., provided multiple data on two objectives of the review and hence, their findings were categorized and repeated under supplementary Table 1 and 2**

1. [↑](#footnote-ref-1)
