# Supplementary material for: Barriers to screening, diagnosis and management of hyperglycaemia in pregnancy in Africa: a systematic review
Source: Int Health. 2021 Aug 25;14(3):211–21. doi: 10.1093/inthealth/ihab054 (PMC9070469; doi:10.1093/inthealth/ihab054)
Supplement: ihab054_Supplemental_File [file ihab054_supplemental_file.zip › Supplementary Table 3.docx]

Supplementary Table 3: Summary of experiences of women and healthcare professionals regarding GDM care in alphabetical (n=3)

| Authors/year/  Country | Aim of the study | Key findings | Main gaps and recommendations |
| --- | --- | --- | --- |
| (Mensah *et al*., 2019)^(27)^  Ghana | Describe the experiences of women regarding the nursing management they received after GDM being diagnosis; and the perceptions of nurse-midwives on the nursing management of GDM | - Worry about the stress of living with diabetes throughout lifetime - Anxiety of delivery outcome for subsequent pregnancies - Fear of risk for future diabetes - Confusion upon a positive GDM diagnosis - Need for referral for psychological support. | In addition to the medical treatment, social, psychological aid, family support and the spiritual wellbeing of women with GDM are essential in the therapeutic process. Health workers must provide care that is culturally sensitive to the customs and beliefs of pregnant women. |
| *(Mensah* et al.*, 2017)*^(31)^  Ghana | Describe pregnant women’s experiences of being diagnosed and living with GDM | - Women expressed mixed feelings about unknown pregnancy outcomes Anxiety prior to the test - Sad and surprised upon been diagnosed - Fear of the unknown outcome of GDM. - In adjusting, some women kept secret their condition - Lamented over lack of attention and sympathy from health professionals and required support from the family. | GDM and the process of detection and management poses some psychological challenges to pregnant women. While capacity building promises to be a cornerstone in managing the condition, there is need for health professionals to be sensitive to these concerns and incorporate them into GDM management |
| *(Muhwava* et al.*, 2019)*^(33)^  South Africa | To explore women’s lived experiences with GDM and the feasibility of  sustained lifestyle modification | - There was anxiety and confusion when referred to higher level of care - Frustration and dissatisfaction with GDM care due to lack of counselling to help women clear their confusion - Discomfort when attempting to engage in a physical activity. - Women who have family history of GDM navigated through pregnancy more easily as they received guidance from previously diagnosed relations. - Some women ensured a healthy lifestyle due to concern for they and their baby’s health. | Besides effective counselling and patient engagement, support from health professionals and family members and creating enabling environment for physical activity are important aspects of GDM management.  While health professionals across Africa remain inadequate to deliver the relevant GDM services, engaging significant others as treatment supporters will promote compliance and improve experiences of pregnant women during GDM care. |

**^Abbreviations: OGTT, Oral glucose tolerance test, GDM, gestational diabetes mellitus, DIP, diabetes in pregnancy, FBG, fasting blood glucose.^**

**^NB, Mensah et al., 2019 provided findings on barriers to management of GDM and was therefore repeated in supplementary Table 2^**
